# Cohort Selection for Clinical Trials From Longitudinal Patient Records: Text Mining Approach

**DOI:** 10.2196/15980

**Published:** 2019-10-31

**Authors:** Irena Spasic, Dominik Krzeminski, Padraig Corcoran, Alexander Balinsky

**Affiliations:** 1 School of Computer Science & Informatics Cardiff University Cardiff United Kingdom; 2 School of Psychology Cardiff University Cardiff United Kingdom; 3 School of Mathematics Cardiff University Cardiff United Kingdom

**Keywords:** natural language processing, machine learning, electronic medical records, clinical trial, eligibility determination

## Abstract

**Background:**

Clinical trials are an important step in introducing new interventions into clinical practice by generating data on their safety and efficacy. Clinical trials need to ensure that participants are similar so that the findings can be attributed to the interventions studied and not to some other factors. Therefore, each clinical trial defines eligibility criteria, which describe characteristics that must be shared by the participants. Unfortunately, the complexities of eligibility criteria may not allow them to be translated directly into readily executable database queries. Instead, they may require careful analysis of the narrative sections of medical records. Manual screening of medical records is time consuming, thus negatively affecting the timeliness of the recruitment process.

**Objective:**

Track 1 of the 2018 National Natural Language Processing Clinical Challenge focused on the task of cohort selection for clinical trials, aiming to answer the following question: Can natural language processing be applied to narrative medical records to identify patients who meet eligibility criteria for clinical trials? The task required the participating systems to analyze longitudinal patient records to determine if the corresponding patients met the given eligibility criteria. We aimed to describe a system developed to address this task.

**Methods:**

Our system consisted of 13 classifiers, one for each eligibility criterion. All classifiers used a bag-of-words document representation model. To prevent the loss of relevant contextual information associated with such representation, a pattern-matching approach was used to extract context-sensitive features. They were embedded back into the text as lexically distinguishable tokens, which were consequently featured in the bag-of-words representation. Supervised machine learning was chosen wherever a sufficient number of both positive and negative instances was available to learn from. A rule-based approach focusing on a small set of relevant features was chosen for the remaining criteria.

**Results:**

The system was evaluated using microaveraged F measure. Overall, 4 machine algorithms, including support vector machine, logistic regression, naïve Bayesian classifier, and gradient tree boosting (GTB), were evaluated on the training data using 10–fold cross-validation. Overall, GTB demonstrated the most consistent performance. Its performance peaked when oversampling was used to balance the training data. The final evaluation was performed on previously unseen test data. On average, the F measure of 89.04% was comparable to 3 of the top ranked performances in the shared task (91.11%, 90.28%, and 90.21%). With an F measure of 88.14%, we significantly outperformed these systems (81.03%, 78.50%, and 70.81%) in identifying patients with advanced coronary artery disease.

**Conclusions:**

The holdout evaluation provides evidence that our system was able to identify eligible patients for the given clinical trial with high accuracy. Our approach demonstrates how rule-based knowledge infusion can improve the performance of machine learning algorithms even when trained on a relatively small dataset.

## Introduction

### Background

**Figure 1:**
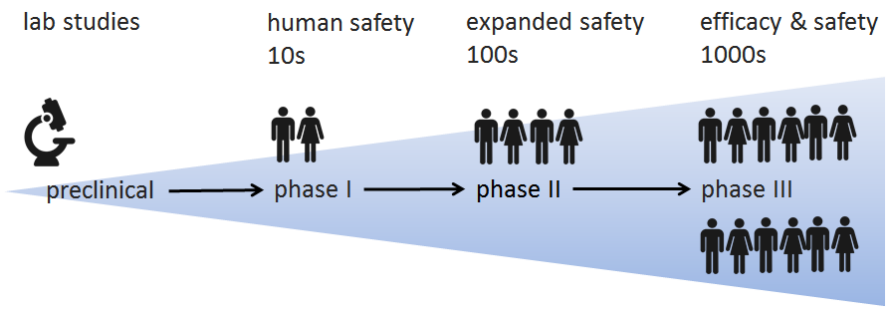
The three premarketing phases of a clinical trial.

Clinical trials are medical research studies focusing on a specific health intervention. They involve human participants to generate data on safety and efficacy as any new health intervention needs to comply with the Hippocratic Oath: “First, do no harm!” With this principle in mind, clinical trials leading up to regulatory approval are typically divided into 3 phases, each involving a significantly higher number of patients (see Figure 1). Phase I aims to answer the following question: Is the intervention safe? The first few healthy participants are given very low doses of the treatment and are monitored closely. If there are no major side effects, the dose is iteratively increased until an effective dose whose possible side effects that are deemed acceptable is reached. Phase II involves patients to determine whether the new intervention works or not. In other words, it assesses its efficacy while continually monitoring the side effects. Finally, in addition to safety and efficacy, phase III also tests the efficiency of the intervention by comparing it with other available interventions. When introducing control groups of participants, clinical trials need to ensure that they are as similar as possible to be able to attribute any findings to the interventions studied and not some other factors. Therefore, each clinical trial defines the eligibility criteria that describe characteristics that must be shared by all participants.

Patient recruitment is universally recognized as a key determinant of success for clinical trials, yet they commonly fail to reach their recruitment goals [1]. Almost a fifth of trials were terminated because they failed to recruit enough participants [2], with less than one-fifth managing to reach their recruitment targets within the proposed time frames [3]. Eligibility represents a major clinical domain barrier to participation [4]. The eligibility criteria are often criticized for being too narrow, thus having a negative impact on recruitment rates and also the generalizability of findings. A stakeholder survey of the barriers to patient recruitment and possible solutions revealed identification of eligible patients using medical records and hospital-based registries and other databases as the key method to improve recruitment [5]. Unfortunately, the complexities of the eligibility criteria do not allow them to be translated directly into readily executable database queries. Instead, they require careful analysis of information contained in the narrative sections of medical records. Manual screening of medical records is time consuming, thus negatively affecting the timeliness of the recruitment process. Text mining has a potential to provide a technical means for unclogging this bottleneck.

### Related Work

The problem of matching the eligibility criteria against their electronic medical records (EMRs) can be framed using a variety of natural language processing (NLP) tasks depending on the type and level of automation expected. In the context of decision making, automation can be applied to 4 classes of functions: information acquisition, information analysis, decision selection, and decision implementation [6]. In our scenario, we focused on a clinician as a human operator who, given a collection of EMRs and a set of eligibility criteria, needs to decide which patients should be recruited to a given clinical trial. In this context, we can think of information acquisition as identification of information relevant to the eligibility criteria. This task can be automated by means of information retrieval (IR) or information extraction (IE).

IR can be applied to both structured and unstructured components of the EMRs to retrieve relevant records or their parts. The usability of any IR system depends on two key factors: system effectiveness and user utility [7]. A test collection of 56 topics based on patient statements (eg, signs, symptoms, and treatment) and inclusion/exclusion criteria (eg, patient’s demographics, laboratory test, and diagnosis) can be used to evaluate the effectiveness of IR for cohort selection [8]. The utility of IR systems can be improved by designing an intuitive visual query interface easily used by clinical researchers [9]. Both utility and effectiveness depend on how well the system incorporates domain-specific knowledge. An ontology can be used to support term disambiguation, term normalization, and subsumption reasoning. Most studies mapped textual elements to concepts in the Unified Medical Language System (UMLS) for normalization with few studies discussing the use of semantic Web technologies for phenotyping [10]. For instance, the UMLS hierarchy can be used to expand a query searching for cancer to other related terms (eg, neuroblastoma and glioma). However, using such a broad hierarchy for unsupervised expansion can introduce many irrelevant terms, which can be detrimental to eligibility-screening performance [11]. This problem can be reduced by using the UMLS to bootstrap creation of custom ontologies relevant to the problem at hand. For example, to identify patients with cerebral aneurysms, a domain-specific ontology was created by querying the UMLS for concepts related to the locations of aneurysms (eg, middle cerebral artery or anterior communicating artery), other clinical phenotypes related to cerebral aneurysms (eg, saccular aneurysm or subarachnoid hemorrhage), associated conditions (eg, polycystic kidney disease), and competing diagnoses (eg, arteriovenous malformation) [12]. Where available, other relevant systems can be used to inform the development of domain-specific ontologies. For instance, the Epilepsy Data Extraction and Annotation uses a novel Epilepsy and Seizure Ontology, which is based on the International League Against Epilepsy classification system as the core knowledge resource [9].

The complexity of clinical sublanguage may require new language modeling approaches to be able to formulate multilayered queries and customize the level of linguistic granularity [13]. This approach to IR incorporates the output of other NLP systems to represent a document or a query using multiple aligned layers consisting of tokens, their part of speech, named entities with mappings to external knowledge sources, and syntactic dependencies among these elements. Other IR efforts focused on directing a clinician’s attention toward specific sentences that are relevant for eligibility determination [14]. This is achieved by segmenting the natural language description of eligibility criteria into individual sentences, analyzing them further to identify domain-specific concepts, and using them to identify sentences in the EMRs that make references to these concepts. This approach is designed to work with categorical data but falls short when numerical data need to be interpreted. For instance, 5 numerical values are needed to diagnose a metabolic syndrome [15]. Of these values, 3 (triglycerides, high-density lipoprotein cholesterol, and elevated fasting glucose) are stored in the laboratory information system, and as structured data are readily available for querying and comparison with referent values. However, in some systems, 2 values may be hidden in the narrative notes (elevated waist circumference and elevated blood pressure). Traditionally, IR approaches are based on the bag-of-words (BoW) model, which represents each document as an unordered collection of features that correspond to the words in a vocabulary for a given document collection. Therefore, by design, IR approaches will be ineffective when it comes to dealing with continuous variables. Conversely, IE based on simple regular expressions can be used to extract numerical values from text and make them amenable for further analysis and interpretation [15-18].

However, the technical feasibility of the IE process does not mean that all relevant attributes are necessarily documented in a single source as the previous example illustrates. For example, a study on case-finding algorithms for hepatocellular cancer discovered significant differences in performance between 2 types of documents (pathology and radiology reports) [19]. It also revealed a significant difference between the narrative reports and coded fields. This raises an important aspect of the completeness of information recorded in an EMR [15]. It has been established that case finding by the International Classification of Diseases, Ninth Revision (ICD-9) coding alone is not sufficient to reliably identify patients with a particular disease or risk factors [20-22]. A few studies contrasted the utility of structured and unstructured information, with the NLP approaches usually demonstrating better results [19,23-28]. In particular, the use of ICD-9 codes for patient phenotyping demonstrated markedly lower precision (or positive predictive value) [19,24,26]. This finding is compatible with a hypothesis that ICD-9 codes are designed for billing purposes and as such may not capture the nuances of phenotypic characteristics in terms of information completeness, expressiveness, and granularity [23].

The analysis of the strengths and weaknesses of both data sources together with practical experiments has led to a consensus that clinical narratives should be used in combination with structured data for eligibility screening [19,23,25,26,28]. Therefore, data fusion is a key component of the information acquisition step in eligibility screening. It should by no means be limited to these 2 modalities of data. For example, clinical electroencephalography (EEG) is the most important investigation in the diagnosis and management of epilepsies. A multimodal patient cohort retrieval system has been designed to leverage the heterogeneous nature of EEG data by integrating EEG reports with EEG signal data [29]. Though evidently important, data fusion techniques are beyond the scope of this study. Here, we focused exclusively on reviewing the methods used to mine clinical narratives for the purpose of eligibility screening. However, the awareness of the need for data fusion can help the reader realize the existence of an externally imposed upper bound on expected performance of text mining approaches.

We have thus far discussed the role of IR and IE in the context of information acquisition. The clinician is still expected to review the retrieved information to decide who satisfies the eligibility criteria. Text mining can be used to support this process by automating information analysis and decision selection by means of feature extraction and text classification, respectively. Two NLP systems tailored to the clinical domain are most often used to extract rich linguistic and semantic features from the narrative found in EMRs: Medical Language Extraction and Encoding (MedLEE [30]) [16,23,25] and clinical Text Analysis and Knowledge Extraction System (cTAKES [31]) [9,11,12,16,18,19,32,33]. They model the semantics by mapping text to the UMLS or a custom dictionary if required. Clinical text analysis needs to make fine-grained semantic distinctions as medical concepts may be negated, may describe someone other than the patient, and may be referring to time other than the present [13]. MedLEE and cTAKES can not only identify concepts of interest but can also interpret their meaning in the context of negation, hedging, and specific sections. Both systems can also perform syntactic analysis to extract linguistic features such as part of speech and syntactic dependencies. Abbreviations are some of the most prominent features of clinical narratives. Unfortunately, both MedLEE and cTAKES demonstrated suboptimal performance in abbreviation recognition [34], which may require development of bespoke solutions [16,35].

Once the pertinent features have been extracted, they can be exploited by rule-based or machine learning approaches. A review of approaches to identifying patient cohorts using EMRs revealed that out of 97 studies, 24 described rule-based systems; 41 used statistical analyses, data mining, or machine learning; and 22 described hybrid systems [10]. A minimal set of rules is sufficient to accurately extract highly standardized information from the narratives [15]. Their development requires iterative consultation with a clinical expert [26]. Nonetheless, a well-designed rule-based system can achieve good performance on cohort selection even with a small training dataset [36], which remains a problem associated with supervised machine learning approaches. When relevant concepts can be accurately identified from clinical text, both rule-based and machine learning approaches demonstrate good performance, albeit it is slightly in favor of machine learning [25,33].

A variety of supervised machine learning approaches have been used to support cohort selection, including support vector machines (SVMs) [22,25], decision trees [22], Repeated Incremental Pruning to Produce Error Reduction, random forests [25], C4.5 [33], logistic regression (LR) [25,28], naïve Bayesian (NB) learning [22,37], perceptron [37], conditional random fields [19], and deep learning [29,38]. Unfortunately, few studies report systematic evaluation of a wide range of machine learning algorithms, thus offering little insight into the optimal performance of machine learning for cohort selection [39]. Another issue associated with supervised learning is that of imbalanced data. The number of positive examples will typically vary significantly across the eligibility criteria. The data used for the 2018 National Natural Language Processing Clinical Challenge (n2c2) shared task on cohort selection for clinical trials provide a perfect illustration of this problem [18,36,38]. Yet, few approaches tackled this issue with different sampling approaches. Instead, they may resort to using machine learning approaches generally perceived to be the most robust for imbalanced data, for example, SVMs [40,41].

Our review of related work illustrates the ways in which the eligibility screening process can be automated. One study reported that the time for cohort identification was reduced significantly from a few weeks to a few seconds [16]. Others reported the workload reduction with automated eligibility screening around 90% [42] achieved a 450% increase in trial screening efficiency [11]. Most recently, the patient screening time was reduced by 34%, allowing for the saved time to be redirected to activities that further streamlined teamwork among the clinical research coordinators [43]. The same study showed that the numbers of subjects screened, approached, and enrolled were increased by 14.7%, 11.1%, and 11.1%, respectively. In this study, we aimed to illustrate the complexity of the eligibility screening problem and propose a way in which this task can be automated.

## Methods

### System Overview

In this paper, we describe Cardiff Cohort Selection System (c2s2) [44], an open-source NLP system that, given a longitudinal patient record, performs binary classification against 13 eligibility criteria for a clinical trial. For each criterion in turn, the system determines whether a patient meets or does not meet a given criterion. The eligibility criteria were predefined by the organizers of the 2018 n2c2 shared task (see Table 1) that aimed to answer the question whether NLP systems can use narrative medical records to identify patients eligible for clinical trials.

**Figure 2:**
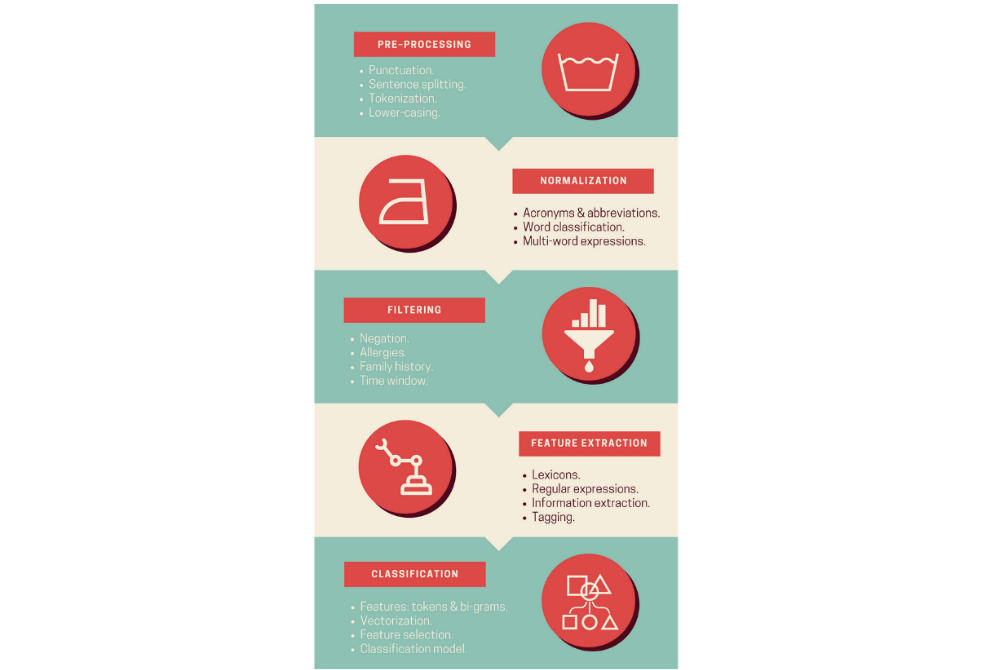
System architecture.

For the majority of criteria, a record needs to contain the supporting evidence for the corresponding patient to meet a given criterion, otherwise the criterion is considered *not met* (eg, if glycated hemoglobin [HbA_1c_] value is 4.7 or missing, then the criterion HBA_1c_ is not met). The only 2 exceptions are the criteria concerning a patient’s ability to speak English and make their own medical decisions, which are assumed to be *met*, that is, the evidence of the contrary needs to be identified to overturn this assumption. Our system is designed to find and tag such evidence in text using a rule-based approach. A text classifier was trained on the tagged text for each criterion that had a sufficient number of both positive and negative representatives to learn from. Overall, the system consists of 5 modules whose functionality is outlined in Figure 2.

The input to the system is a longitudinal patient record distributed as a single UTF-encoded text file, which contains multiple records generated across various health care encounters. Each individual record represents either a discharge summary or a correspondence between health care professionals [45,46]. Their content may cover patient demographics, progress notes, problems, prescribed medications, vital signs, past medical history, immunizations, laboratory data, and radiology reports. Individual records start with a line formatted as *Record date: YYYY-MM-DD* and are arranged in the ascending order by the record date. Other than that, there are no other restrictions on the format of individual records. Indeed, they may reflect a variety of different styles.

**Table 1:** Description of the eligibility criteria, as provided in the annotation guidelines used for the National Natural Language Processing Clinical Challenge shared task.

ID	Criterion	Time period	Default
ABDOMINAL	Intra-abdominal surgery, small or large intestine resection, or small bowel obstruction	Any	Not met
ADVANCED-CAD	Advanced artery disease	Present	Not met
ALCOHOL-ABUSE	Alcohol use exceeds weekly recommended limits	Present	Not met
ASP-FOR-MI	Use of aspirin to prevent myocardial infarction	Any	Not met
CREATININE	Serum creatinine is above the upper limit of normal	Any	Not met
DIETSUPP-2MOS	Use of dietary supplements (excluding vitamin D)	Past 2 months	Not met
DRUG-ABUSE	Drug abuse	Any	Not met
ENGLISH	Speaks English	Any	Met
HBA_1c_	Glycated hemoglobin value is between 6.5 and 9.5	Any	Not met
KETO-1YR	Diagnosed with ketoacidosis	Past year	Not met
MAJOR-DIABETES	Major diabetes-related complication	Any	Not met
MAKES-DECISIONS	Able to make decisions for themselves	Present	Met
MI-6MOS	Myocardial infarction	Past 6 months	Not met

### Preprocessing

In addition to standard preprocessing operations (see Figure 2), special consideration is given to punctuation. Its use in clinical narratives proved to affect the results of text segmentation algorithms developed for general language [47]. On one hand, clinical narratives commonly use punctuation as means of abbreviation (see Table 2 for examples). Such use of punctuation may easily be misinterpreted as a sentence terminator. For instance, phrases such as “q. Sunday,” “vit. D,” and “Dr. Harold Nutter” feature a period followed by an uppercase letter, a pattern that is commonly exploited in both rule-based and machine learning approaches to split sentences. Segmentation errors can propagate onto the subsequent stages of text processing, resulting in the loss of syntactic dependencies between related words and consequently their contribution to the overall semantics. For example, incorrectly splitting a sentence within the phrase “vit. D” would effectively erase this mention of “vitamin D,” a named entity of direct relevance to the eligibility criterion DIETSUPP-2MOS (see Table 1). To prevent parsing errors of this type, pattern-matching rules were developed to identify and remove punctuation used in such contexts before performing sentence segmentation (see Table 2 for examples).

**Table 2:** A selection of rule-based punctuation removal examples.

Rule target	Input	Output
Prescription	q. a.m. q. Sunday tab.	qam q Sunday tab
Vitamin	vit. D MVit.	vit D MVit
Personal title	Dr. Harold Nutter Harold Nutter, Ph.D.	Dr Harold Nutter Harold Nutter, PhD
Shorthand x	hx. of migraines sx. of depression Rx. for cpap	hx of migraines sx of depression Rx for cpap
Species name	E. coli C. diff H. pylori	E coli C diff H pylori

Clinical narratives also feature prevalent use of short formulaic statements such as field:value combinations (eg, *Substance abuse: none*) and itemized lists (see Textbox 1 for an example).

Such statements are not commonly terminated by means of punctuation. When used consecutively, this can often result in independent statements being incorrectly grouped together in a single sentence. Their intersentential co-occurrence may later be easily confused with relatedness. Consider, for instance, amalgamating the above itemized list into a continuous sequence “*s/p cerebral infarction myocardial scan normal blood pressure today 190/108.”* It could lead to incorrectly recognizing *infarction* as a *myocardial* one and the *blood pressure* as *normal*, when in fact, the *infarction* is *cerebral,* and the *blood pressure* is *abnormally high*. Acting preemptively, we perform document layout analysis to identify itemized lists and insert punctuation where appropriate before performing sentence segmentation. Consequently, this will enforce independent fragments to be interpreted as separate sentences.

Finally, to streamline subsequent text analysis, we use pattern-matching rules to fully expand enclitics and special characters. For example, *couldn't* is expanded to *could not*, whereas *con't* is expanded to *continue*. This will later simplify identification of negated expressions. Similarly, to prune the number of IE rules, we lexicalized a relevant set of special characters. For example, *BUN/Cr ratio is* >*20* would become *BUN/Cr ratio is greater than 20*.

**An example of assessment recorded as an itemized list.**

- *s/p cerebral infarction*
- *myocardial scan normal*
- *blood pressure today 190/108*

### Normalization

Text normalization is performed with a similar intent: to simplify subsequent text analysis. It involves mapping of a selected subset of words and phrases onto their representatives, which can be either a preferred synonym or a hypernym (see Table 3 for examples). Special consideration is given to acronyms and abbreviations as they are known to have a major impact on retrieval of relevant information. First, disambiguation is performed for a small subset of abbreviations of direct relevance for the given classification tasks. Examples include *ca* (*calcium* vs *cancer*), *mg* (*magnesium* vs *milligram*), and *CR* (*creatinine* vs *controlled release*). A context-sensitive approach is used to select an appropriate interpretation. For example, if CR is used in combination with words such as *tablet* or *capsule*, then it is assumed to refer to *controlled release*.

**Table 3:** Examples of text normalization.

Example	Surface forms	Normalized form	Relevance
1	mom, father, sister	family member	filtering
2	FH, FHx, FamHx	family history	filtering
3	whiskey, vodka, beer	alcohol	ALCOHOL-ABUSE
4	Lantus, Humalog, NPH	insulin	MAJOR-DIABETES
5	DM2, DMII, NIDDM	diabetes mellitus 2	MAJOR-DIABETES
6	CRRT, CRRTX	continuous renal replacement therapy	MAJOR-DIABETES
7	ARF	acute renal failure	MAJOR-DIABETES
8	CKD	chronic kidney disease	MAJOR-DIABETES
9	BB, bblocker, betablocker	beta blocker	ADVANCED-CAD
10	ECG, EKG	electrocardiogram	ADVANCED-CAD
11	ICD	implantable cardioverter defibrillator	ADVANCED-CAD
12	CVD	cardiovascular disease	ADVANCED-CAD
13	MI, heart attack	myocardial infarction	MI-6MOS, ASP-FOR-MI, ADVANCED-CAD
14	STEMI	ST elevation myocardial infarction	MI-6MOS, ASP-FOR-MI, ADVANCED-CAD
15	ASA, ECASA	aspirin	ASP-FOR-MI

Other acronyms and abbreviations of interest are then expanded using a bespoke lexicon (>500 entries) developed specifically for this task. To bootstrap the lexicon construction, the raw training data were used to analyze frequently occurring words. Orthographic features (uppercase typeset, eg, *STEMI*, or the use of punctuation, eg, *q.a.m.* or *r/o*) and spelling checker (eg, *inpt*) were used to identify potential acronyms and abbreviations as *unknown* words that are also relatively short. Medical expertise was used to identify the corresponding full forms. Simple Concordance Program [48] was used to verify manually whether the proposed full forms apply across the majority of contexts within the training data to enable the use a context-free approach for acronym and abbreviation expansion.

The only acronym exempt from expansion was *CCB*. In fact, all occurrences of *calcium channel blocker* were replaced by the corresponding acronym. The reason behind this decision is the fact that both *calcium* as a supplement and *calcium channel blocker* often occur in similar context (eg, medication list). As one of the eligibility criteria was concerned with dietary supplementation (see DIETSUPP-2MOS in Table 1), this reduced the risk of interpreting the latter mention of *calcium* as a supplement.

To illustrate the extent to which text normalization can simplify its subsequent analysis, we can use examples provided in Table 3. For example, by replacing the surface forms in Example 1 by their hypernym and expanding abbreviations in Example 2, we can simply use the occurrence of the word *family* to filter out sentences or the whole sections that refer to family members. Consider, for example, the original text given in Textbox 2 and its normalized counterpart in Textbox 3.

**An original example of family history.**

*FH: Mom w/ PM at age 50, died of MI at 71. Father w/ EtOH, HTN. Sister w/ 4 miscarriages.*

**A normalized example of family history.**

*Family history: Family member with pacemaker at age 50, died of myocardial infarction at 71. Family member with alcohol abuse, hypertension. Family member with 4 miscarriages.*

By filtering out references to family members, we are effectively removing the mentions of *myocardial infarction* and *alcohol abuse* that do not apply to the given patient. Consequently, we can use the remaining references to *myocardial infarction* and *alcohol abuse*, if any, as evidence for eligibility criteria MI-6MOS and ALCOHOL-ABUSE (see Table 1). Similarly, by mapping alcoholic beverages in Example 3 to their hypernym, the subsequent analysis related to the eligibility criterion ALCOHOL-ABUSE (see Table 1) can simply focus on any mention of the word *alcohol*. Examples 4 and 5 show that 2 keywords, *insulin* and *diabetes*, can be used to look for evidence of diabetes. Once unpacked from the corresponding acronyms (Example 5), the word *diabetes* becomes accessible to text analysis. Similarly, words *renal* and *kidney* become visible after expanding acronyms in Examples 5-7. Knowing that diabetes is a major risk factor for kidney disease, we can subsequently use close occurrences of the word *diabetes* to either of the words *renal* or *kidney* as evidence for the eligibility criterion MAJOR-DIABETES (see Table 1). Similar to lexical analysis, morphological analysis can be used to identify features relevant to the given eligibility criteria. Normalized forms in Examples 10-14 related to ADVANCED-CAD (see Table 1) incorporate a morpheme *cardi(o)*, which signifies that these medical concepts are related to the heart, which can be affected by coronary artery disease.

### Filtering

Once the text has been regularized by means of preprocessing and normalization, information not directly relevant to the given classification tasks is filtered out. We focus on 4 types of such information:

1. negation, for example,* ruled out for MI by enzymes*
2. family history, for example,* mother died at age 62 of a heart attack*
3. allergies, for example,* Allergies: aspirin—GI upset*
4. time window, for example,* records older than the last 6 months*

Removal of such information simplifies subsequent classification by allowing the use of a BoW approach. For example, by not considering the first 2 examples, the risk of misclassifying a patient as having a *myocardial infarction* is reduced. Similarly, by removing the third example from consideration, the risk of misclassifying a patient as one taking *aspirin* to prevent *myocardial infarction* is also reduced. Finally, as some of the eligibility criteria were time dependent (namely, ALCOHOL-ABUSE, DIETSUPP-2MOS, KETO-1YR, MAKES-DECISIONS, and MI-6MOS—see Table 1 for definitions), we identified dates of individual medical records to extract the ones relevant to the given time windows and stored them separately for use by the corresponding classifiers.

We used a set of regular expressions, which are available from the c2s2 GitHub repository [42], to identify the 4 types of information considered. Regular expressions used to identify negation are based on the NegEx algorithm for identifying negated concepts in clinical notes [49].

### Feature Extraction

Thus far, we reduced the noise and lexical variability in the data by means of filtering and normalization. This is expected to improve the performance of a supervised classifier. Another action that stands to improve the classification performance when trained on a relatively small dataset is that of reducing dimensionality of a BoW representation by aggregating related features into a single representative. In its simplest form, feature aggregation can be achieved by abstracting words into semantic classes. Where domain ontology is available, such abstraction can be automated by exploiting its taxonomic structure. The Semantic Network of the UMLS can be used to automatically abstract words into semantic types. However, as examples given in Table 4 illustrate, the UMLS semantic types are too broad in the context of eligibility criteria described in Table 1. For example, abstracting Examples 1-4 into pharmacologic substance would dilute rather than distil relevant information. A finer-grained abstraction tuned for the given eligibility criteria would be more appropriate (see the last 2 columns in Table 4), but it would also incur some knowledge engineering overhead. However, the widespread availability of Web resources that summarize information pertaining to health and well-being can greatly reduce such overhead. We defined a total of 8 abstraction categories and assembled the corresponding lexica using online resources (see Table 5).

**Table 4:** Examples of word abstraction.

Example	Surface forms	Semantic type	Abstraction	Relevance
1	marijuana, heroin, ecstasy	Pharmacologic substance	Illicit drug	DRUG-ABUSE
2	beta blocker, nitroglycerin, CCB	Pharmacologic substance	Heart medication	ADVANCED-CAD
3	crestor, advicor, compactin	Pharmacologic substance	Statin	ADVANCED-CAD
4	vitamin C, calcium, primrose oil	Pharmacologic substance	Supplement	DIETSUPP-2MOS
5	turmeric, green tea, cinnamon	Food	Supplement	DIETSUPP-2MOS
6	vodka, beer, wine	Food	Alcohol	ALCOHOL-ABUSE

**Table 5:** Rule-based feature extraction.

Tag	Feature	Extraction^a^	Examples^b^
MEDRX	Prescription instructions	Regular expressions	po q4h prn
KIDMED	Kidney medication	Lexicon (221 entries)^c^	Thymoglobulin
BRPMED	Blood pressure medication	—^d^	Avapro
HRTMED	Heart medication	—	Plavix
HRTTRT	Heart treatment	Regular expressions	Re*catheteriz*ation
HRTISC	Heart ischemia	Regular expressions	Electro*cardio*gram demonstrated *ischemic* changes
HRTANG	Angina	Regular expressions	Chest wall heaviness
HRTCAD	Any of the HRT tags above + explicit references to CAD	Regular expressions	Given his extensive cardiac history
ASPFMI	Aspirin for heart problems	Regular expressions	Start on heparin *HRT*MED and *aspirin* and take to *HRT*TRT catheterization laboratory
SPLMNT	Supplement (strong evidence)	Lexicon (67 entries) + regular expressions	Ibuprofen 800 mg *MED*RX *potassium* chloride 10 meq *MED*RX lasix 20 mg *MED*RX
DFCNCY	Supplement (weak evidence)	Lexicon (27 entries) + regular expressions	*Iron deficien*cy anemia
MNTCAP	Mental capacity	Regular expressions	Increasing *disorientat*ion and visual *hallucinat*ions
DRGADD	Substance abuse	Lexicon (17 entries) + regular expressions	History of *cocaine* abuse
NOENGL	Does not speak English	Lexicon (66 entries) + regular expressions	An *Indonesian speaking* 85-year-old male
ALCABS	Alcohol abuse	Lexicon (7 entries) + regular expressions	*Alcoholism* 10 years ago
ALCSTP	Stopped drinking alcohol	Regular expressions	Alcoholism 10 *years ago*
KETACD	Ketoacidosis	Regular expressions	Ketones positive
KIDDAM	Kidney problems	Regular expressions	*Worse*ning *renal* dys*function*
DMCMPL	Diabetic complications	Regular expressions	*Diabet*es mellitus related retino*pathy*/neuro*pathy*
ABDMNL	Abdominal surgery or small bowel obstruction	Regular expressions	Gastric *laparo*scopic bypass surgery
HIGHCRT	High creatinine	Regular expressions + information extraction	Blood urea nitrogen/*creatinine* of 21/*1.7*
GLYHMG	Glycated hemoglobin in a given interval	Information extraction	*HbA*_1c_ one month ago was *6.7*

^a^All lexicons and regular expressions are available from the c2s2 GitHub repository [44].

^b^Italic typeset is used to indicate the types of text features targeted by lexicons and regular expressions.

^c^KIDMED, BRPMED, HRTMED are organized into a single lexicon of 221 entries.

^d^Not applicable.

Once the BoW representation is passed onto a supervised classifier, the context of individual words will be lost. For instance, blood tests frequently feature essential minerals such as calcium, potassium, and iron, which can also be prescribed under the same names as supplements. The BoW approach will take these names out of context, keeping their frequency as the only information about them. Conversely, simple pattern analysis can be used to differentiate between the 2 types of context. For example, we can model prescription instructions using regular expressions (see Table 5) and tag this information in text in the form of a token (eg, MEDRX) that is lexically distinguishable from other tokens. We can subsequently apply another regular expression to find mentions of essential minerals in the close proximity to the MEDRX token and tag such mentions using another special-purpose tag (eg, SPLMNT). When we now apply the BoW approach, the token SPLMNT, treated as any other text token, will represent a feature that preserves relevant contextual information. Supervised machine learning algorithms can then take advantage of such a feature in combination with the standard BoW features. Regular expressions are used to embed a total of 18 context-sensitive features into text (see Table 5).

Regular expressions can be used to model categorical references to information relevant to the given eligibility criteria. For example, regular expressions can be used to link the word *creatinine* with a stem *elev*-in the phrase *a mildly elevated creatinine* and use it as an indication for meeting the eligibility criterion CREATININE (see Table 1). However, knowing whether serum creatinine is above the upper limit of normal in a phrase such as “blood urea nitrogen and creatinine ratio of 40 and 1.0 respectively” requires not only extracting the correct numerical value (1.0) but also comparing it with the reference value (1.5). Two eligibility criteria, CREATININE and HBA_1c_, require extraction of numerical information and its subsequent analysis, as indicated in Table 5. As before, the outcome of such context-sensitive analysis is embedded back into the text for further exploitation by supervised machine learning.

Overall, a total of 22 tags described in Table 5 were chosen so that they can be lexically and orthographically distinguishable from other words upon their imputation into the processed text. The corresponding features are extracted incrementally in the order given in Table 5 and, when appropriate, used to support extraction of other features. For example, knowing that heparin is a heart medication (indicated by the tag HRTMED—see Table 5) can be used to infer that, when aspirin is taken together with heparin, it is likely to be used as prophylaxis for the prevention of cardiovascular events such as myocardial infarction (indicated by the tag ASPFMI—see Table 5).

### Classification

**Figure 3:**
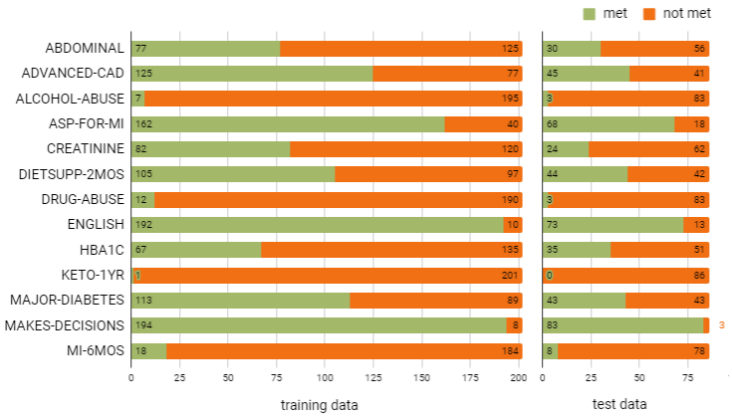
Distribution of class labels.

This module consists of 13 binary classifiers, 1 for each eligibility criterion (see Table 1). The distribution of class labels in the training data informed the choice of a classification method. Supervised machine learning was chosen wherever a sufficient number of both positive and negative instances were available to learn from (see Figure 3). A rule-based approach focusing on a small set of relevant features was chosen for the remaining criteria (see Table 6). The corresponding classification rules were based on a relevant set of manually engineered features described earlier in Table 5. Each rule was defined as a function of these features and a threshold value that maximizes the class separation, both chosen manually. The only exception was associated with the criterion MI-6MOS, where the final rule was induced from the training data in the form of a decision tree using a manually selected set of features.

**Table 6:** Features used in rule-based classification.

ID	Features
ALCOHOL-ABUSE	ALCABS, ALCSTP
DRUG-ABUSE	DRGADD
ENGLISH	NOENGL
KETO-1YR	KETACD
MAKES-DECISIONS	MNTCAP
MI-6MOS	BRPMED, HRTMED, HRTTRT, HRTISC, HRTANG, HRTCAD, ASPFMI

Note that the numerical values used in criteria CREATININE and HBA_1c_ were also extracted using a rule-based approach. However, in a longitudinal report, different values may be reported at different time points. In the absence of clear guidelines, we used machine learning on top of IE to determine automatically from the training data how to deal with such cases.

A machine learning approach was used for all other criteria. According to the *no free lunch* theorem [39], there is no universally best learning algorithm. In other words, the performance of machine algorithms depends not only on a specific computational task at hand but also on the properties of the data that characterize the problem. To compare the performance of different algorithms, we used 10–fold cross-validation experiments. We chose a representative algorithm from 4 major categories: function-based learning, regression analysis, probabilistic learning, and ensemble learning. Specific algorithms chosen were SVM with radial basis function kernel, LR, NB classifier, and gradient tree boosting (GTB), respectively. In our experiments, we used implementations of the first 3 algorithms in scikit-learn, an open-source Python library for data analysis and modeling [50]. Experiments with GTB were performed using XGBoost, an open-source software library that implements a gradient boosting framework for Python [51]. All experiments were performed with the default parameter values.

We trained all classifiers using single words and/or bigrams as features with and without feature selection based on L1 regularized linear SVM. The overall performance was statistically indistinguishable across different types of features used. Therefore, we opted for a simple BoW approach with feature selection for efficiency reasons. To evaluate the impact of the class imbalance on the classification performance, we balanced the training data using random undersampling and oversampling with default parameters from scikit-learn [50].

**Figure 4:**
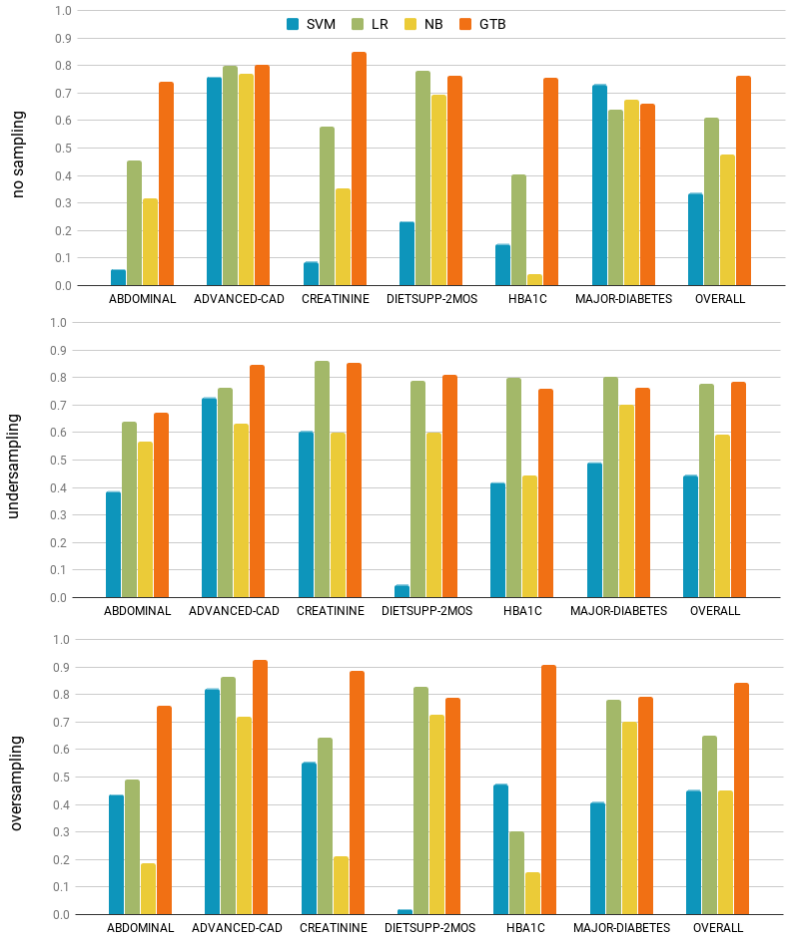
Summary of cross-validation results. SVM:support vector machines; LR: logistic regression; NB: naïve Bayesian; GTB: gradient tree boosting; HBA_1c_:glycated hemoglobin.

Figure 4 summarizes the performance in terms of microaveraged *F* measure. Overall, GTB demonstrated the most consistent performance. Its performance peaked when oversampling was used to balance the training data. GTB is an ensemble classifier over a set of simple decision trees, which are varied according to specific parameter settings (learning rate and maximum tree depth). Having chosen GTB as the learning method, we optimized its parameters by performing grid search on learning rate (0.001-0.5) and maximum tree depth (2-10) using the oversampled training data. The learning rate of 0.02 and maximum depth of 10 were chosen for the holdout evaluation described in the next section.

## Results

The results of classification experiments on previously unseen test data are summarized in Table 7. The evaluation results were calculated using a script released by the organizers of the 2018 n2c2 shared task. We used the best results from 3 related studies as the baseline. They used rule-based [36], hybrid [18], and hierarchical neural network (HNN) [38] approaches. We interpret the results for each classifier separately.

**Table 7:** Detailed holdout test results.

ID	Met^a^				Not met^a^				Overall		Baseline^b^		c2s2^c^
	P^d^ (%)	R^e^ (%)	F^f^ (%)	P (%)		R (%)	F (%)	F (%)		F (%)		System	Rank
ABDOMINAL	64.86	80.00	71.64	87.76		76.79	81.90	76.77		90.64		Rules	4
ADVANCED-CAD	83.02	97.78	89.80	96.97		78.05	86.49	88.14		88.14		c2s2	1
ALCOHOL-ABUSE	22.22	66.67	33.33	98.70		91.57	95.00	64.17		*89.70*		Hybrid	2
ASP-FOR-MI	87.67	94.12	90.78	69.23		50.00	58.06	74.42		77.34		HNN^g^	2
CREATININE	80.00	83.33	81.63	93.44		91.94	92.68	87.16		89.75		Rules	2
DIETSUPP-2MOS	78.85	93.18	85.42	91.18		73.81	81.58	83.50		89.53		Hybrid	4
DRUG-ABUSE	40.00	66.67	50.00	98.77		96.39	97.56	73.78		*92.55*		Hybrid	2
ENGLISH	91.25	100.00	95.42	100.00		46.15	63.16	79.29		97.66		Hybrid	4
HBA_1c_	100.00	82.86	90.62	89.47		100.00	94.44	92.53		93.82		Rules	2
KETO-1YR	0.00	0.00	0.00	100.00		100.00	100.00	50.00		*50.00*		All	1
MAJOR-DIABETES	85.00	79.07	81.93	80.43		86.05	83.15	82.54		86.02		Hybrid	2
MAKES-DECISIONS	97.62	98.80	98.20	50.00		33.33	40.00	69.10		*74.40*		HNN	2
MI-6MOS	33.33	50.00	40.00	94.59		89.74	92.11	66.05		*87.59*		Rules	4
Overall^h^ (microaveraged)	83.97	91.29	87.47	93.54		87.86	90.61	89.04		91.11		Hybrid	4

^a^The binary classification task involves 2 classes (*met* and *not me*t). The results are provided for each class separately and then combined into the overall F value.

^b^The best results from 3 related studies are used as the baseline. They are named after the approach they used: rules [34], hybrid [17], and HNN [36]. The baseline results in italics were calculated on the basis of at most eight positive examples, which account for less than 10% of the test data.

^c^c2s2: Cardiff Cohort Selection System.

^d^P: precision.

^e^R: recall.

^f^F: *F* measure.

^g^HNN: hierarchical neural network.

^h^The overall values provided in the bottom row have been microaveraged across the 13 classifiers.

The best results marked with an asterisk in Table 7 were calculated on the basis of at most 8 positive examples, which account for less than 10% of the test data. This makes it impossible to differentiate between random and statistically significant outcomes, thus making it difficult to generalize the findings. The most extreme example is that of KETO-1YR, which had no positive examples in the test data. The results of all 4 systems were identical with no classification errors. Again, given that the training data contained only 1 positive example, the best classification strategy would be the majority rule, which would achieve the same result. Similarly, ALCOHOL-ABUSE, DRUG-ABUSE, and MAKES-DECISIONS had only 3 positive examples in the test data. On these classes, the 4 systems achieved average precision, recall, and *F* measure of 58.43%, 65.46%, and 59.38% with standard deviations of 40.11%, 36.51%, and 37.48%, respectively, again illustrating the difficulty of generalizing these findings. Finally, MI-6MOS had 8 positive examples. The rule-based system achieved the best performance followed by HNN. At 40.00%, the remaining 2 systems achieved a modest *F* measure on the *met* class, but they did differ in the way they balanced precision and recall. Overall, no obvious pattern could be noticed in the classification performance on this class.

All 4 systems achieved similar performance for HBA_1c_ and ASP-FOR-MI. On the *met* class, all 4 systems achieved maximal precision on HBA_1c_ with recall in the 80s, resulting in an *F* measure just below or just above 90%. Conversely, on the *met* class, all 4 systems achieved almost perfect recall on ASP-FOR-MI with precision in the high 80s, resulting in an *F* measure over 90%. Given the consistently high performance, we infer that the 2 eligibility criteria are semantically tractable in the sense that they lend themselves to being modeled computationally.

The rule-based approach performed best against the following eligibility criteria: ABDOMINAL and CREATININE. For ABDOMINAL, recall was in the 80s on the *met* class with no significant variation across the systems. However, the 2 machine learning approaches demonstrated markedly lower precision than the rule-based approach: 60s versus 90s. Further experiments are needed to determine whether more training data would help reduce the number of false positives. In reality, the cost and time associated with data annotation imposes an upper bound on the amount of training data available. Given the *F* measure is in high 80s, rule-based approaches could be a preferred option for narrowly defined eligibility criteria, which can be mapped to explicit references in text. We can observe similar results for CREATININE. The rule-based approach performed best with an *F* measure in the 80s on the *met* class, followed by our own approach with comparable performance. Although we used machine learning, the key feature used by the classifier was in fact extracted using a rule-based approach. This is consistent with our previous recommendation.

Conversely, broader eligibility criteria, which require some reasoning over multiple references made across the discourse, may require a machine learning approach to model the complexities of target classification problems. MAJOR-DIABETES is one such example where major complications may not be restricted to a finite class of signs and symptoms. In addition, such complications may be mentioned without an explicit reference to diabetes. This requires complex analysis of the wider context. Neural networks can be used to model nonlinearity in text. Not surprisingly, the HNN approach achieved the best results in this case. In particular, the robustness of this approach is reflected in achieving a recall of over 90% on the *met* class. The rule-based approaches demonstrated lower recall. Our own approach demonstrated the lowest recall as we also used a rule-based approach to extract pertinent features. However, our use of machine learning on top of such features resulted in the second highest precision on the *met* class.

Another example of this type of problem is ADVANCED-CAD. As expected, both machine learning approaches performed better than the other 2, with overall *F* measure in the 80s and 70s, respectively. In particular, our approach significantly outperformed all others in both precision and recall (see Table 8). We attribute such a performance to a suitable combination of rule-based feature extraction and supervised classification. By examining Table 5, we can see that the majority of features are related to advanced cardiovascular disease either directly (eg, HRTMED, HRTTRT, HRTISC, HRTANG, HRTCAD, and ASPFMI) or indirectly (eg, BRPMED and DMCMPL). Our approach demonstrates the degree to which domain knowledge infusion can improve the performance of machine learning when trained on a relatively small dataset. However, it does not require comprehensive knowledge elicitation. We simply used online resources and simple corpus analysis to inform the development of the corresponding lexica and regular expressions following the same approach used successfully in previous shared tasks [52,53].

**Table 8:** Detailed holdout test results for ADVANCED-CAD.

System	Met			Not met			Overall
	P^a^ (%)	R^b^ (%)	F^c^ (%)	P (%)	R (%)	F (%)	F (%)
c2s2^d^	83.02	97.78	89.80	96.97	78.05	86.49	88.14
Hybrid	74.55	91.11	82.00	87.10	65.85	75.00	78.50
Rules	67.80	88.89	76.92	81.48	53.66	64.71	70.81
HNN^e^	77.36	91.11	83.67	87.88	70.73	78.38	81.03

^a^P: precision.

^b^R: recall.

^c^F: *F* measure.

^d^c2s2: Cardiff Cohort Selection System.

^e^HNN: hierarchical neural network.

## Discussion

Ideally, supervised learning performs best when large training datasets with a reasonable class balance are available to extrapolate a classification model while minimizing overfitting. As we can see from the data (see Figure 3), this was not the case in this particular study. This is likely to be the norm in practice rather than the exception. When structured data are available to support certain eligibility criteria, there is no need for analyzing the unstructured text data. When such a need does exist, the use of supervised learning requires manual annotation of text data, which requires clinical expertise. The cost and time associated with this activity naturally imposes an upper bound on the amount of training data available. This limited amount of training data will immediately exclude approaches such as deep learning, which, in theory, could be used to extract complex relationships between words using long- and short-term memory. Therefore, the remaining choices include rule-based classification and supervised learning. Clinical trials are plagued by insufficient recruitment rates. On average, 86% of trials fail to recruit a sufficient number of patients, 85% of trials overrun because of insufficient recruitment, 37% of sites do not meet their recruitment targets, and 20% fail to recruit any patients [54]. Even when sufficient numbers are initially recruited, the problem of 30% dropout rate remains. Not surprisingly, 30% of phase III trial terminations are because of recruitment failures. Owing to these recruitment concerns, one would naturally opt for supervised learning approaches as they are more robust than rule-based approaches in terms of recall. In other words, it would help identify a much larger pool of patients to potentially recruit. However, the limited amount of training data will prevent the use of longer n-grams as it would lead to document representation vectors that are long and sparse, a combination prone to overfitting. This leaves the BoW approach as the most plausible option. To compensate for the loss of context, manual feature engineering can be used to model complex relationships between words. This represents a practical compromise between rule-based and machine learning approaches. This study provides a practical example of such a hybrid approach. The development of our system incurred less than 2 person-months, while achieving performance that could boost the recruitment. The system is expected to reduce clinicians’ workload in line with the estimates reported by other studies [11,16,42,43].

## Abbreviations

BoW: bag-of-words
c2s2: Cardiff Cohort Selection System
cTAKES: clinical Text Analysis and Knowledge Extraction System
EEG: electroencephalography
EMR: electronic medical record
GTB: gradient tree boosting
HBA_1c_: glycated hemoglobin
HNN: hierarchical neural network
ICD-9: International Classification of Diseases, Ninth Revision
IE: information extraction
IR: information retrieval
LR: logistic regression
MedLEE: Medical Language Extraction and Encoding
n2c2: National natural language processing Clinical Challenge
NB: naïve Bayesian
NLP: natural language processing
SVM: support vector machine
UMLS: Unified Medical Language System
